# M. Cazenave on Syphilitic Eruptions of the Skin

**Published:** 1847-04

**Authors:** 


### ART. II.

*Traité des Syphilides, ou Maladies Vénériennes de la Peau, précédé de Considérations sur les Syphilis, son Origine, sa Nature, &c. Accompagné d'un Atlas in-folio, contenant douze Planches dessinées d'après Nature, gravées et coloriées.* Par ALPH. CAZENAVE, Médecin de l'Hôpital Saint Louis, &c. &c.—*Paris,* 1844.

*A Treatise on the Syphilitic Eruptions of the Skin, preceded by general Remarks on the Nature and Origin of Syphilis; accompanied by an Atlas of twelve Plates, coloured and engraved, and drawn from Nature.* By ALPH. CAZENAVE, Physician to the Hospital of Saint Louis, &c. &c. —*Paris,* 1844. 8vo, pp. 630.

IT has been aptly observed by a French writer, that we might frequently economise time and trouble, by satisfying ourselves on two points, before undertaking the perusal of a new medical work. The facts to be ascertained are these:—Has the author had sufficient opportunity for studying the subject which he has undertaken to describe, and if so, was he mentally qualified for the task? If we test by this ordeal the claims on our attention of the author, whose work we are about to review, we shall find that they are worthy of our best consideration.

Biett was the first to describe separately the syphilitic eruptions, and to draw the attention of medical men to that interesting class of diseases. In fact, he made their study peculiarly his own, and, although he never published a distinct work on the subject, he promulgated his views through the medium of lectures, which were afterwards published by his pupil, M. Cazenave, in his excellent 'Manual of Diseases of the Skin.' M. Cazenave enjoyed the friendship of Biett for nearly twenty years, and has been attached to the Hospital of St. Louis for the same period, in different capacities; hence we may view the 'Traité des Syphilides' in a great measure as the expression of Biett's opinions on the venereal eruptions. Indeed, M. Cazenave states it was originally intended that this work should be a joint production, and laments that a long illness, and subsequently death, deprived him of the valuable co-operation of so sound and conscientious an observer as his late friend and master, Biett. But, if we have occasion to regret. that the eminent dermatologist who had the care of the Hospital of St. Louis for a long series of years has not himself written on a subject which he understood so well, we have the satisfaction to know that the work of his pupil and successor, M. Cazenave, contains a faithful exposition of his views and extensive experience in this branch of dermal pathology.

M. Cazenave's work is divided into two parts. The first is occupied with the history and general pathology of syphilis; and the second contains an elaborate account, illustrated with cases and coloured plates, of the various syphilitic eruptions. It is with the latter division that we shall be chiefly occupied in the present article, but as some of the author's views on the primary disease are peculiar, it is proper that the reader should be made acquainted with them in the first instance.

### I. PRIMARY SYPHILIS.

The author sets out with the following propositions, which he considers are supported by the past history of syphilis, and by the observations of himself and others: 1. There is a syphilitic disease. 2. It has existed from the remotest antiquity. 3. It is the result of a special poison which infects the system; there is but one virus. 4. Syphilis is contagious. 5. It is hereditary. 6. It is announced by various primary symptoms; it occasions secondary symptoms. 7. Mercury is still the best remedy that can be employed in the treatment of that disease. It would be foreign to our present purpose to follow M. Cazenave through all the *questiones vexatio* arising out of the foregoing propositions; but it is due to the author to state a few of his arguments in favour, of the antiquity of the disease, of one virus, and of the mercurial treatment.

I. The author considers that syphilis is not a disease of modern origin, and essays to prove that proposition from the works of the earliest writers —from the Leviticus of Moses down to the writers at the close of the fifteenth century, the period which has generally got the credit of being distinguished by the birth of the *morbus gallicus*.

Under the generic name of gonorrhœa, says M. Cazenave, the ancients have confounded all manner of venereal infection, and hence has arisen the idea that the syphilitic disease, according to the general acceptation of that term, was altogether unknown to them. Hippocrates, in describing the *morbus femineus* of the Scythians, mentions the occurrence of ulcers of the pudenda. Celsus recommends (De Medicina, l. vi, cap. 18) the operation of phymosis in a case of a rebellious chancre on the corona. Galen describes buboes and ulcerations resulting from disease of the genital organs.

Oribasius, Ætius, Paulus Ægineta, and several other writers of the same period, describe the disease more distinctly; but the question of antiquity is not solely dependent on medical authority for support. It is further corroborated by historical writers, as for example by Pliny and Josephus. The former relates the case of a woman who was drowned in the lake of Como, because her husband was attacked in his private parts with an incurable disease (*maritus ex diutino morbo circà velanda corporis ulceribus putrescebat*), and Josephus, in describing the death of Herod, states that the genitals were in a state of putrefaction, (*ipsa quoque verenda putrefacta scatebant.*) With regard to the secondary symptoms, or syphilitic eruptions, M. Cazenave considers that they were confounded with lepra, elephantiasis, mentagra, lichen, &c., a mistake which, by the by, is by no means uncommon even at the present day. The author also quotes several of the Arabian writers in support of his views, and then proceeds to discuss the famous epidemic of the fifteenth century, and the supposed American origin of the disease.

The author alleges that the epidemic of 1494 was not syphilis, nor did syphilis, or that epidemic, originate in America. The disease which ravaged Europe towards the close of the fifteenth century, was one of those plagues which history tells us has visited the world from time to time in all ages. It was of a typhoid and not syphilitic character; but the frightful immorality of the time, and its results, and a remarkable similarity between certain symptoms of the epidemic and of the venereal disease, will, according to M. Cazenave, account for the erroneous views regarding their identity promulgated by the writers of that period.* At the time alluded to, both diseases evidently coexisted; and the symptoms of the syphilitic malady acquired a violent intensity under the influence of the epidemic; but if the former were merely the result, or continuation of the latter, as alleged, "how is it," asks the author, "that on the cessation of the epidemic, the venereal disease continued to exist independent of the former, of which it was, in point of fact, a terrible auxiliary, but not a consequence?" M. Cazenave regards the notion of the American origin of syphilis as a fable invented by a Spanish writer, Oviedo, in 1518, nearly twenty years after the commencement of the epidemic, in order to justify or palliate the frightful crimes committed by the Spaniards in America, and which excited the indignation of Europe. In support of this view he cites a host of authors, and some interesting cases; but we must refer the reader to the original work for the details.

II. M. Cazenave is decidedly opposed to the doctrine of a double virus as regards the venereal diseases, or, in other words, he holds the opinion that chancre and gonorrhœa are results of the same specific poison.

"Being placed under favorable circumstances," says the author, "for observing the development of the secondary symptoms of syphilis, I am in a position to state that in a great number of cases, the general infection of the system has originated in a gonorrhœa (blennorrhœa); and if my opinion, with the proofs which I shall adduce by and by, shall be deemed insufficient for establishing this fact, I may

* M. Heusinger states, in his remarkable work, 'Recherches de Pathologie Comparée,' "that most of our domestic animals are subject to contagious discharges and ulceration of the generative organs; they are transmissible by coitus, and exhibit a resemblance to syphilitic diseases. In the opinion of M. Heusinger, they are nearly in the same condition as the syphilitic affections of man were before the close of the fifteenth century."—Rev.

invoke the testimony of M. Baumes, who asserts that syphilitic diseases of the skin are very frequently the result of a blennorrhagic discharge, and altogether independent of syphilis. I hope to be able to show that blennorrhagia, like chancre, may produce that morbid condition called constitutional syphilis."

M. Cazenave endeavours, with much earnestness of purpose, to establish this very unorthodox view of the theory of syphilis. He denies that gonorrhœa is the result of simple inflammation of the mucous membrane, and is at issue with the doctrine of Balfour, Duncan, and Benjamin Bell, who were the first supporters of the non-syphilitic character of the urethral discharge. He reprobates the unscientific and illogical argument so frequently put forth by the partisans of this doctrine, namely, that if a patient should contract syphilis from a female labouring under gonorrhœa, and apparently no other disease, still there must have been syphilitic virus present, *however it might have been concealed*, or else the patient could not have contracted that disease. This, the author alleges, is an easy way of answering opponents, but will not bear the test of scrutiny.

The "*chancre larvé*" of M. Ricord comes under this category, and is pronounced by M. Cazenave to be a sophistical figure of speech, a loophole for retreat when obliged to explain how it sometimes happens that inoculation with the muco-purulent matter of blennorrhagia produces chancre.

The author ridicules M. Ricord's idea of a "visionary" chancre, by which he can account for those cases in which syphilitic sores are produced by contact with gonorrhœal matter, and also his *post hoc propter hoc* conclusion that if inoculation with matter supposed to be syphilitic does not produce a chancre, the virus was not venereal; but if it did succeed, it must have been genuine syphilitic pus.

We freely admit that the doctrines of M. Ricord are open to objection on many points, that his inferences are not always correct, nor his analogies complete; but however plausible the counter-statement put forth by M. Cazenave may be, it does not appear to us to be conclusive of the validity of his own theory of a single virus. But it is only fair to let our author explain in his own terms the unity of the syphilitic poison. Nothing is more common, says the author, than to meet with cases where a man has taken syphilis from a female, who upon examination proved to have no chancre, but merely a muco-purulent discharge,—at least M. Cazenave meets with such cases constantly. M. Ricord objects to this, that there was a chancre, but it was overlooked; a statement which is easier made than proved; and the author prefers to view this fact as proving that the muco-purulent matter of blennorrhagia may produce a chancre, rather than to adopt the sophism of his antagonist. In support of this view, he cites the authority of Hunter, the results of experiments conducted by M. Castelneau, and some even conducted by M. Ricord himself. M. Castelneau succeeded in producing the true syphilitic pustule from the matter of blennorrhagia, and M. Ricord succeeded in six cases at least in obtaining the same result. It is a fallacy to say that blennorrhagia is not syphilitic, because inoculation with its matter will not always produce like results, for there are other symptoms, the "pustule plate," for example, which cannot be inoculated, and it is not only a syphilitic symptom, but frequently a primary symptom. It is by no means uncommon to see two individuals infected from the same source, and yet one will have a gonorrhœa and the other a chancre.

But the advocates of a double virus, says the author, will occasionally have need of a triple poison to explain some of their own views: for example: Vigarous relates a case in which six young men, intimately united by friendship, had connexion one after the other with the same woman, who infected all with the venereal disease. The first and fourth (according to the order in which they presented themselves for treatment) had chancres and buboes; the second and third gonorrhœa; of the last two, one had a chancre, and the other a single bubo. This girl must have had a triple virus, chancre, bubo, and gonorrhœa. But how shall we explain the singular fact that all these individuals, being placed in the same position, under circumstances alike favorable to all, were infected by different diseases? Why have they not all had chancres, buboes, and gonorrhœa? The partisans of the double virus will perhaps say that individual susceptibility and different poisonous matter may, in some sort, explain the problem.

M. Cazenave follows up his critical exposition of what he considers fallacious in M. Ricord's doctrine, with great energy, but at the same time without evincing any of that asperity or partisanship which too frequently disfigures and destroys the value of scientific discussion. Although he denies the truth of M. Ricord's axiom that chancre only can produce chancre, and that chancre is the only characteristic symptom of genuine syphilis, he does so by opposing facts against experiments, which are not infallible, and merely considers M. Ricord the victim of a theory which stakes the whole doctrine of syphilis on the point of the lancet.

"I am aware," says Cazenave, "that M. Ricord has made numerous experiments, that he has been able to try them at will, and that on the great arena where he has taken so prominent a position by his zeal and industry, and the *éclat* of his researches, he has been able to collect all the elements necessary for rational conviction. Nevertheless, I am compelled to say that he is deceived; he has only regarded syphilis in one of its phases, and, carried away by his love for experiments, he has forgotten that the theory of venereal infection can never be reduced within the limits of a mere experiment."

III. With regard to the general treatment of syphilis, our author is again at issue with M. Ricord. This practitioner, it is well known, considers syphilis to be a local disease at the commencement, and hence concludes, that if you can arrest it in this stage by cauterization or other means, you preserve the constitution from syphilitic taint. But M. Cazenave considers the postulate and its corollary to be equally erroneous, and the treatment by cautery irrational, if not injurious. The author does not found his objection on the common occurrence of bubo, swelled testicle, chordee, &c., after the sudden arrest of blennorrhagia, nor of a simple syphilitic sore being converted into a phagedenic or gangrenous ulcer by the imprudent use of the cautery. He opposes it on higher grounds: he views chancre and blennorrhagia as merely the symptoms, the expression of a general infection, and concludes that their disappearance is no proof of the eradication of the virus they represent. We might as well attempt to cure rabies by cauterizing the bitten part after the manifestation of the symptoms of hydrophobia. But the most injurious result likely to occur from M. Ricord's doctrine is that it may prevent the adoption of a rational and general treatment, by means of which the constitution may be relieved from syphilitic taint, and, consequently, all the secondary symptoms prevented.

M. Cazenave's experience goes to prove that the cauterizing treatment is decidedly favorable to the development of the sequelæ of syphilis, and especially of the venereal eruptions. The author does not advocate the use of mercury as a *specific* for syphilis; far from it; but he regards it as the best remedy we possess for enabling the constitution to rid itself of the general taint, and as a prophylactic against secondary symptoms. With regard to the accidents alleged to result from the administration of mercury, it is enough to say that these accidents occur to patients labouring under the disease who never took mercury, as proved by Cullerier, Biett, and Ricord; and, on the other hand, they are scarcely ever met with in individuals who have been under the influence of that remedy, but who have not had syphilis;—a fact established by the joint researches of Biett and the author. M. Cazenave selected 153 syphilitic patients, whom he observed with great care, and took every precaution to avoid deceit. These observations have led him to the following conclusions:

"Mercury is not a specific for syphilis; and however carefully it may be administered, it will not always prevent a relapse; the primary symptoms of the disease may disappear under the influence of an antiphlogistic treatment, but the mercurial treatment is the most powerful means we possess for aiding a constitutional reaction against the syphilitic virus."

### II.—SYPHILITIC ERUPTIONS.

Although the venereal disease appears to have first shown itself in Europe under the form of cutaneous eruptions, and the earliest writers on syphilis confined their descriptions of that complaint to a pustular affection of the skin; still, until the early part of the nineteenth century there was no attempt made to arrange and classify the syphilitic cutaneous diseases under a distinct head. However, about that period these eruptions were grouped together for the first time, and described under the name of *syphilides;* but as this classification was formed without any reference to the elementary characters of the diseases, distinct varieties were confounded together, and species established on characters that were altogether secondary and insignificant. Such was the history of the syphilides when Biett first turned his attention to the subject, and his admirable and unrivalled essay, published in M. Cazenave's Manual, bears testimony to the value and importance of his researches into the history of that interesting class of diseases.

From the close of the fifteenth to the beginning of the nineteenth century the syphilitic eruptions were described under the vague general term of *pustules,* and even the writings of the learned Astruc, John Hunter, and B. Bell are characterized by the same vagueness of description as those of the authors who described the venereal eruptions of 1493. Alibert was the first to substitute the more correct and significant term "*syphilide*" for that of pustule, the propriety of which Biett at once recognized, and subsequently adopted it in his lectures on the syphilitic eruptions. Moreover, this sound practitioner saw the necessity of studying this group of diseases apart, according to their different forms, their relation with the primary symptoms, and the actual state of the patient at the time; and following out this view, he classified the syphilides according to their elementary lesions, and established with a clearness and precision hitherto unknown, the striking and well-defined characters of the syphilitic exanthemata, the syphilitic vesiculæ, the syphilitic pustulæ, the syphilitic tuberculæ, papulæ, and squamæ. Under these six groups every variety is included, and each variety has its own distinct description.

These are the views promulgated by M. Cazenave in the work now under consideration.

By the term *syphilide* the author means every cutaneous eruption developed under the influence of the venereal virus, and characterized by the elementary lesions of the simple eruptions, but possessing a certain specific physiognomy, which is altogether unique. The syphilides are primary or consecutive. In the first instance they accompany chancre or blennorrhagia, or, as more frequently happens, immediately succeed the sudden removal of these lesions by cauterization or other revulsives. But the *consecutive* syphilides are by far the most frequent. These manifest themselves after the total disappearance of the primary symptoms, and long after the patient fancies himself free from all syphilitic taint. They invariably assume a chronic character, and present three different orders of symptoms, which may be described as—1, symptoms which are *common* to all varieties; 2, those which are *special;* and, 3, *concurrent* symptoms.

*Common symptoms.* Amongst the various symptoms which—if we may use the term—individualize the syphilides, the *colour* of the eruption, its form, the tint of the adjoining skin, and the nature of the squamæ, scabs, and cicatrices are the most remarkable. All the syphilitic eruptions are stamped by a peculiar tint or colour, unique of its kind, more or less distinct according to circumstances, but the constant and invariable attendant of every variety, whatever may be its form. It is extremely difficult to give a precise definition of this colour, and even Fallopius, who points out the importance of this symptom, in a diagnostic point of view, admits the difficulty, for he says, "*Color non potest explicari, non enim est ruber, non albus, non pallidus.*" This colour, then, which it is impossible to define by a single term, varies from a *coppery red* to a brownish gray, and it is the dull, grayish, obscure, intermediate shades which constitute what is called the "syphilitic tint." This copper colour is the most constant and remarkable symptom of the syphilides. It is of itself sufficient to identify the disease, and is invariably present in every variety and form of these eruptions without exception. The change of colour is produced by a vitiated secretion of the chromatogenous apparatus, and not by an altered condition of the capillary rete, as alleged by some writers. It is analogous to the abnormal colorations of the ephilides and the pigmentary nævi; it does not disappear under the pressure of the finger, and the depth of the colour depends in great measure upon the degree of injection of the cutis; for example, when there is much sanguineous congestion present the syphilitic tint is masked altogether, and according as this condition subsides it becomes gradually more pronounced.

The shade of colour is also regulated by the nature of the eruption and the temperament of the patient; thus, in a young sanguineous subject, with a fine, white skin, attacked with roseola, or certain forms of lichen syphilitica, the "tint" partakes somewhat of the colour of the adjoining surface, by reason of the capillary injection, but it is always duller than that of the surrounding skin, and as the general redness disappears, it assumes its natural coppery violet colour. In persons in the decline of life, with a dry, shrivelled skin, the tint is still duller, and of a violet red shade. In persons of a bilious habit, whose skin is naturally brown, the syphilitic tint presents remarkable shades; it is, in general, scarcely at all red, but rather of a dark gray colour, approaching a brown, which is gradually blended with that of the surrounding tissue; and, finally, in cachectic individuals, the colour of the eruption is livid, and even old cicatrices in these cases present a peculiar blueish appearance.

The syphilides have a remarkable tendency to assume a *circular form.* This disposition is not confined to isolated patches of limited extent, but may be observed in cases where the eruption is considerably diffused. In the latter instance, although the circle may not be complete, we may always find detached patches, showing a distinct tendency to form a ring. This circular form, however, is not invariably pathognomonic of the disease, for there are some varieties in which it does not appear, and, on the other hand, it occurs in certain cutaneous diseases, which are not special; as, for example, herpes, lepra, lichen, &c.; but again, it should always be remembered that certain eruptions which do not, in their simple form, show this peculiarity, invariably do so when they become syphilitic. For example, the tubercular and pustular syphilides show it in a remarkable degree, and even the horny syphilide, which we sometimes see in the palms of the hands, also evinces this peculiarity. The face, above all other regions of the skin, seems to be particularly predisposed to this form of the eruption, and hence the origin of the term *corona veneris.*

The syphilitic eruptions are further characterized by their tedious chronic progress through their various stages. They are slow in being developed, the process of suppuration is difficult, and cicatrization is long in being accomplished; in short, each phase is longer of being completed than another, and this very chronicity forms an important feature of the disease. The anatomical characters are also *sui generis.* In the squamous eruptions the scales are thinner and drier than those of the simple forms of these diseases. They are likewise much smaller, and never cover the patch completely, but form a whitish fringe or list (*lisière*) around it, to which Biett attached much importance as a diagnostic. The scabs are formed slowly, and the papular elevations of psoriasis and lepra syphilitica are frequently observed without any. The syphilitic incrustations are thick, greenish, sometimes black, hard, furrowed, and very adherent. They always indicate a certain amount of destruction of the tissues beneath. These scabs sometimes cover ulcerated surfaces, and then they are softer, broader at their base, surrounded by a flabby copper-coloured rim, or they may repose on a cicatrized base, in which case they are dry, shrivelled, horny, and uneven at their base, and by gradual destruction disclose a cicatrix beneath, into which they seem to penetrate by a kind of mammillated projection.

These considerations lead us to speak of a very important character common to all the syphilides, with the exception of the exanthemata and squamæ. We allude to the remarkable tendency which these diseases evince to destroy the tissues in which they are produced, as shown by the indelible cicatrices which they leave behind. The syphilitic ulcerations belong exclusively to the vesicular, pustular, and tubercular species. They are round, more or less deep, grayish or very red at the base, with swollen and sharp cut edges. The round form of the ulcerations is always present; it is especially marked in those which occur in ecthyma. It is not so clearly defined in the tubercular syphilides; in the serpiginous ulcer, for example, the borders are certainly circular, but the ring is less regular in form, and, moreover, it is not the result of a single tubercle, but of several, and of repeated destruction of the tissues. It is a remarkable fact, that this destructive process is frequently accomplished without ulceration or a breach of surface, and we may observe the curious phenomenon of the formation of an indelible cicatrix, without the slightest wound or breach in the continuity of the skin. This singular process of destruction is particularly observable in the papular and tubercular syphilides, and seems to be the result of degeneration of the tissue in the first instance, and of absorption of it afterwards.

The syphilitic cicatrices present several characters which belong also to the lesions they have succeeded. Thus they are generally round, and more or less depressed; when recent they are of a bronze colour, and are sometimes slightly prominent, and the superficial vessels may be seen ramifying beneath the epidermis. At a later period they shrink, the process of absorption apparently going on internally; they lose their violet colour, and become white and still more depressed, their surface, which is now of a dead white hue, is tense or puckered, and sometimes intersected with hard prominent bands. In some cases they are of a blueish white tint from the beginning, and are surrounded by a copper-coloured areola, which gradually subsides, until it becomes lost in the tint of the surrounding skin.

SPECIAL SYMPTOMS. Whatever may be the characters by which the syphilides are known, whatever may be their aspect, they invariably commence in the form of spots or blotches (*taches*), vesicles, blebs, pustules, papulæ, squamæ, or tubercles; hence M. Cazenave proposes to classify and describe them in the following order:

SYPHILIDES {

- Exanthemata

- Vesiculæ

- Bullæ

- Pustulæ

- Papulæ

- Squamæ

- Tuberculæ.

*Syphilitic exanthemata.* These eruptions are characterized by irregularly-shaped blotches, sometimes scarcely raised above the level of the skin, of a coppery red colour at first, which, by and by, passes into a grayish tint, and disappears slowly but never completely under pressure of the finger. They are rarely accompanied by febrile symptoms, are never followed by ulceration or cicatrix, and the only trace that remains after resolution, is a slight stain, which sometimes continues for several months. There are two varieties in this class pretty distinct from each other. In one (roseola) the spots are broad and irregular, and scarcely, if at all, raised above the surface. In the other (erythema papulatum) the patches are more circumscribed, distinctly rounded, and slightly prominent.

Roseola is one of the least common of the syphilitic eruptions. It consists in slight patches or *efflorescences,* more or less diffused, and usually seated on the chest, the neck, the face, and upper extremities ; of a violet or copper colour at first, which finally changes to a gray tint. It is rarely accompanied by febrile disturbance, but it is not unfrequently preceded by dull pains in the limbs, headache, and angina, characterized by a violet-red colour of the mucous membrane of the mouth, velum palati, and pharynx, accompanied by a sensation of heat and difficulty of swallowing. M. Cazenave has even observed in some cases, a peculiar ulceration of the amygdalæ with sharp cut edges and gray base, but never deep or extensive.

*Erythema papulatum* shows itself in the form of patches of very limited extent, not larger than the circumference of a shilling, and frequently less, slightly prominent, round, of a grayish brown tint at first, and subsequently a pale red, which does not disappear under pressure, and terminates by resolution. This eruption appears without premonitory symptoms, and most commonly on the extremities, especially the arms. It is frequently ephemeral ; it appears and disappears continually, and is suddenly transposed from one place to another. Its duration is much shorter than that of roseola, and it is never followed by desquamation. M. Cazenave is of opinion that this eruption is frequently the result of the sudden suppression of blennorrhagia by copaiba, &c., but altogether independent of that medicine, for he has met with the disease in many cases where it was not administered. The author has satisfied himself that both roseola and erythema syphilitica are generally produced by the sudden removal of chancre or blennorrhagia, more especially the latter, by means of revulsives. M. Cazenave here relates several cases illustrative of the primary and consecutive forms of the syphilitic exanthemata. In the latter, the eruptions appeared in intervals of from two months to ten years after the primary disease, and evinced the same characters in each instance.

*Syphilitic vesiculæ.* This species was supposed, until very recently, to be the rarest of all the venereal eruptions. Biett met with only a few cases. But M. Baumes and M. Cazenave agree in stating that syphilitic vesicular eruptions are by no means so rare as supposed, and require merely careful observation to be detected. M. Cazenave distinctly states that they may appear in any of the forms of the simple vesicular disease. Sometimes they manifest themselves in the character of round, globose, detached vesicles, as in varicella ; sometimes in the form of small circular discs, as in herpes ; occasionally in an eruption of numerous vesicles thrown into groups, and disseminated, as in eczema. The vesicles are distinguished from those of the simple forms, by a copper-coloured areola round their base. Contrary to the habit of the syphilides in general, the vesiculæ seldom appear on the face. They are most commonly observed on the neck, chest, and upper extremities. Their duration is also shorter than that of the other species, and varies from two or three weeks to as many months. The author gives several cases illustrative of the different varieties of these eruptions, and amongst others an interesting case of syphilitic varicella, of which there is a well-executed coloured plate in the accompanying atlas. As it will answer the purpose of a detailed description, we shall give this observation in full.

"A young girl, 16 years of age, of healthy constitution, had complained for a few days of heat in the throat, with difficulty of swallowing, anorexia, and irregular fever; a number of small eminences now appeared on different parts of the body, and she entered the Hospital of St. Louis. The eruption was at once seen to be vesicular, and pronounced chicken-pock. It was the sixth day of the eruption; it covered nearly the whole body, and the vesicles were in different stages; some being nascent, others dried up. Biett having examined the patient, discovered a strong resemblance between this eruption and two other cases of syphilitic vesicular eruption which had come under his observation. This diagnosis was soon confirmed by the progress of the disease. The vesicles were small, resting on a broad base, and surrounded by an areola of a vivid copper colour; their progress was slow, and they were unattended by any local symptoms. They gradually faded away, and the fluid was absorbed; but, in some, the contents of the vesicle hardened into a thin scab, which adhered for some time. Every one of them, however, left behind a coppery injection of the skin, which presented all the characters of a syphilitic blotch. In addition to these circumstances, a careful examination of the throat disclosed a round grayish ulcer, with sharp cut edges, &c. The treatment employed was insignificant, as Biett wished to see if any more decisive symptoms would manifest themselves; but the patient left the hospital in a fortnight. After the lapse of a month she was visited at home, when her body was found covered with true syphilitic pustules."

*Syphilitic bullæ.* The bullæ have been observed in the syphilitic form in the only two varieties known of that class, pemphigus and rupia. Syphilitic pemphigus is almost invariably a disease of new-born infants, derived from their parents, and consists in an eruption of one or more bullæ, of irregular size and form, existing at the period of birth, and usually situated on the palms of the hands and soles of the feet. The blebs are soft, contain a sero-purulent fluid, and are surrounded by a violet-coloured areola. Rupia syphilitica is more rarely met with than the preceding variety. In this instance the bullæ are larger, nearly circular, contain a blackish fluid, which dries into a scab of the same colour, of a conical form. They are surrounded by a copper-coloured areola, and terminate in indelible cicatrices of a circular form.

*Syphilitic pustulæ.* The history of the pustular syphilides is, perhaps, more interesting than that of any other class of venereal eruptions; because, under this title, all the syphilitic eruptions were described by former writers, and also on account of the great frequency of some of its varieties. Although every form of the simple pustular diseases may appear on the skin under a syphilitic character, M. Cazenave confines his descriptions to the following varieties as being most commonly met with in practice: 1, a syphilitic pustulo-lenticular eruption; 2, impetigo, in two distinct forms—impetigo non confluens, and a syphilitic pustulo-crustaceous variety; 3, ecthyma. The lenticular form is the most common of the syphilitic pustulæ. It is characterized by an eruption of isolated pimples, about the size of a small lentil, irregularly diffused, slightly prominent, of a peculiar colour, suppurating partially, and terminating in a small cicatrix, of less diameter than the original lesion. It attacks every region of the skin; the face, the back, the chest, the limbs, &c., and varies a little in form according to the region in which it is developed.

On the face, chest and back it corresponds to acne; the pustules are larger, more prominent, and round; they suppurate only partially, and are crowned by an incrustation of some thickness, terminating in a cicatrix with a depressed centre. On the limbs they are flattened especially round the base, which is broader, and less rounded than that of the preceding. They are of a copper colour, and the size of a small lentil. The centre becomes slightly prominent, and we may presently remark a minute collection of pus at the apex, which disappears in the course of a day or two, either by absorption or by the rupture of the pustule. The elementary lesion now assumes a new phase. It is converted into a small copper-coloured papular-like elevation, with a depressed cicatrix at its apex, which is sometimes perforated down to the base of the pimple. According as the disease advances, it loses its pustular appearance, and might be mistaken by a careless observer for a papular eruption; but new pustules are constantly appearing in the vicinity of the old, which will serve to clear up the diagnosis. It is, however, owing to the frequency of errors of this kind, that some writers have supposed that the papular eruption was one of the most frequent of the syphilides, and M. Cazenave has had cases under his care where the disease, strange to say, was mistaken for common itch. The progress of the lenticular eruption is always slow, and it terminates in a minute central cicatrix superimposed on a hardened base.

The simple form of syphilitic impetigo closely resembles syphilitic varicella. The pustules, which are of moderate size, are detached from each other, are preceded by a red copper colour, have not a hardened base, and are formed by an elevation of the epidermis, which covers completely the blotch. This variety has the appearance of a number of small, soft, slightly resistent tumours, pretty close to each other without uniting, and encircled by a reddish areola. It is usually preceded by febrile symptoms. It may appear at once over an extensive surface of the body, the abdomen, buttocks, internal aspect of the thighs, and sometimes on the upper extremities, but seldom on the face. If the pustules are not torn, they may continue without change for several days; the contained fluid gradually coagulates into a brownish scab, larger than the pustule, which, by and by, becomes so thin that you can see a cicatrix beneath. In some rare cases the pustules increase in size, and run into each other, and form a single incrustation.

The pustulo-crustaceous impetigo is a much severer form of the disease than the preceding, and is particularly obnoxious from occurring so frequently on the face. It commences by a redness and tumefaction in the parts about to be attacked, which are soon followed by small collections of matter. These speedily unite, and then the disease consists in one or more large blotches, surrounded by a copper-coloured areola, and covered by slightly raised scabs, irregular in form, greenish, soft to the touch, incurvated at the centre, and flattened at the circumference. These scabs conceal grayish ulcerations, with slightly elevated edges, which secrete a sero-purulent matter. By means of this matter, the incrustations are incessantly renewed until the disease is at length modified, then they dry, shrivel, and fall off, exposing to view an unseemly scar, whose deformity is in proportion to the number of times the scabs have been reproduced. This eruption may appear on several parts at the same time, but it has no tendency to spread to the adjoining parts, and generally acquires at once the size and form it retains throughout its different stages. It attacks the chest, the neck, but more especially the face and forehead, and, unlike the preceding variety, is seldom seen on the lower extremities.

*Ecthyma syphilitica* is characterized by pustules of a much larger size than the preceding varieties, forming small isolated tumours, with indurated bases, containing purulent matter, and terminating speedily in incrustations, which in their turn are succeeded by cicatrices. In some cases the pustules are larger than those of impetigo, but do not exceed the circumference of a sixpence. They are perfectly round, slightly conical, distended by a thick yellowish fluid, are surrounded by a copper-coloured areola, but are not accompanied by induration at the base. These burst in a short time, and are then covered with a brown scab of the same size and form as themselves, slightly adherent, with raised edges, and concealing a superficial ulcer. This variety is generally diffused over a considerable surface of the skin, and occurs frequently on the scalp. Although the pustules are usually scattered, we sometimes, but rarely, may observe them thrown into groups, in which event they terminate in a broad thick scab, not unlike that of the pustulo-crustaceous variety already described. There is another form of syphilitic ecthyma and severer than the foregoing, in which the pustules are considerably larger, and of an oval form. They commence by a violet-coloured blotch, which rises at the centre, and is quickly distended by a thick fluid resembling a mixture of pus and blood. The pustule is encircled by a livid areola, which again is embraced by one of the characteristic copper tint. At the point where the distension of the epidermis ceases, there is a slight puffiness, which gives the pustule a flattened appearance. The coats of the pustule soon give way, and the contained fluid is partly discharged; this coagulates and forms a blackish incrustation, thick at first, but gradually dries into an eschar. This scab, which is exactly the form of the pustule it has succeeded, is more prominent at the centre than at the circumference. If the incrustation be removed at an early period, we shall see beneath a deep ulcer with a grayish base, studded with small red granulations, with sharp cut edges, and close to the latter a whitish line, which is composed of portions of epidermis, separating the scab from the ulcer. If the scab is not interfered with, but allowed to proceed in its course, we may observe it gradually desiccate, and fold upon itself, as it were, by the desiccation of the central portion. The white border mentioned fades and desquamates, and we may now observe the circumference of the incrustation penetrating, so to speak, into the substance of the skin; the scab itself gradually gives way and cracks, until it finally disappears, and is replaced by a round depressed cicatrix of a peculiar violet tint. This eruption occurs most frequently on the extremities, especially the lower, and is sometimes accompanied by a papular disease. The pustular syphilides are most commonly consecutive, but they may be primary symptoms, and syphilis sometimes manifests itself by them alone.

*Syphilitic tuberculæ.* The tuberculæ, which have been invariably confounded with the pustular diseases by former writers, are by far the most frequent of all the venereal eruptions. They appear in the form of small, solid, resistent tumours, containing neither pus nor serum, and more or less prominent. They are sometimes disseminated over a large surface, sometimes, on the contrary, disposed in groups, few in number, and confined within narrow limits. Their size and form is also variable. In some cases they are small, about the size of a pea, round, shining, and of a coppery colour; in others, broad, flattish, spherical, or ovoid, and seem imbedded in the substance of the skin. Sometimes they remain smooth, and shining throughout, sometimes covered with thin scabs: when ulceration takes place, it is succeeded by thick scabs; they occasionally terminate without leaving any other trace than a grayish stain, which disappears in the course of time, but more frequently in an indelible cicatrix, whose size and form depend upon whether it was preceded by ulceration or not. Finally, whatever may be the severity of the disease, it sometimes pursues its course, and corrodes the tissues from without inwards, without extending beyond the parts on which it first appeared, whilst, on the contrary, we not unfrequently meet with cases in which it extends its ravages over a considerable surface, destroying the skin to a greater or less extent in its progress. This frightful disease may appear on any part of the body; but unfortunately, it most frequently occurs on the face, the nose, the ears, the eyebrows, and the scalp. It sometimes is developed slowly and gradually, whilst in other instances it appears suddenly, is accompanied by fever, inflammation of the mucous membrane of the pharynx, velum palati, and tonsils, and pursues its course with great rapidity.

M. Cazenave describes five well-marked varieties of the tubercular syphilitic eruption: 1, cases in which the tubercles are disposed in groups; 2, those in which they are disseminated; 3, a corroding form of the disease; 4, a *serpiginous* variety; 5, one characterized by flattened tubercles. The characters of the first two varieties may be found in the preceding remarks. They are of a milder nature than those which succeed, and the tubercles in some cases are very minute, and resemble papulæ, and rarely suppurate. They are always consecutive, never appearing with the primary disease.

The third variety is very different from the two preceding, and always tends to destructive ulceration. In cases of this kind the tubercles are large, few in number, penetrate deeply into the cutaneous tissue, of a semispherical form, and pointed at the apex. They occur almost invariably on the face, especially the nose and lips, and are sometimes accompanied by considerable tumefaction, in the midst of which they are buried, and can with difficulty be detected. This tumefaction gives way under appropriate treatment in some few instances, and resolution follows, but more commonly this condition appears and disappears until at length the parts are struck with inflammation of an unhealthy character, and ulceration ensues. The tubercles, after remaining stationary and indolent for a considerable time, are suddenly reanimated. The areola, which had almost faded, assumes a more intense hue than ever. The tumours now begin to ulcerate at the apex, and generally follow one of two courses: they may remain soft, scarcely painful, and but slightly ulcerated; or become tense, exceedingly painful, surrounded by an erythematous blush, and the ulceration at the apex extends deeply into the substance of the tubercle. The ulceration is succeeded by a blackish, dry, thick incrustation, which soon falls off, and discloses a deep ulcer with cleanly cut edges, a new scab is formed, falls, and exposes to view a more extensive destruction of the parts, and finally a deep eschar, of a violet colour, of an almost circular form, and as regularly shaped as if cut out by a chisel, terminates the progress of the disease. Thus, in an exceedingly short space of time, a frightful mutilation of the face may ensue, and the alæ of the nose, the lips, the ear, and the eyelid are more or less involved in the destructive ulceration going on around. M. Cazenave gives several interesting cases illustrative of this variety, which we regret want of space will not allow of being related here.

*Serpiginous tubercular eruption.* This variety has a still stronger tendency to destroy the tissues in which it is developed than the foregoing, but it has this peculiarity—that the ulceration is much more superficial, but covers a greater extent of surface than the preceding form, so that, what it loses in depth it gains in length. Indeed, we may sometimes see the whole body covered with indelible and irregularly-shaped cicatrices. The serpiginous eruption manifests itself in the form of large, red, hard, and circular tumours, scattered here and there, few in number at first, and varying from the size of a pea to that of a filbert. They are most commonly observed on the face and trunk, but the author has seen many cases in which they attacked the whole of the cutaneous envelope, with the exception of the palms of the hands and soles of the feet. They are seen not unfrequently on the back of the neck, scalp, and shoulders, and seem to have a predilection for parts covered by hair, as, for example, the temples, eyebrows, anus, genitals.

These tubercles are smooth and shining, of a coppery tint, are never covered by squamæ, and remain indolent and stationary for a long time. Then they become suddenly inflamed from some accidental cause, ulcerate at their apices, the tubercle is completely destroyed, and is succeeded by a thick, hard, conical incrustation, of a black or grayish colour, and very adherent. If this scab is detached before the formation of a cicatrix, it exposes to view a gray superficial ulcer, which is soon covered by another incrustation, not so black or compact as that just described, but always thicker at the centre than at the circumference. When once the process of ulceration is established, the disease advances rapidly, by the development of new tubercles close to the old, or at the edge of the cicatrices. The inflammatory process is renewed here, and this state of things goes on until extensive ulcerations are formed, and destruction of the surrounding tissues, to a greater or less extent, ensues.

While the ulceration is healing at one of its extremities, new tubercles are being incessantly developed at the other, so that we may frequently see in the same patient and at the same time all the characters of the eruption: thus, we may find a crop of salient copper-coloured tubercles scattered round the diseased parts; scabs of various degrees of density, size, and hardness, dry and prominent; grayish ulcerations, with sharp cut edges, some of which are round. others without any regular form, and more twisted in spirals, all of which are intermingled with indelible cicatrices. These, again, are either old, whitish, fibrous-looking eschars, or more recent and copper-coloured, and intersected by superficial vessels. They unite pretty uniformly, but are here and there interrupted by furrows or indentations, which give them a peculiar figured appearance. They are crossed by bands of considerable thickness, which frequently arrest muscular motion, and are studded with small tubercular eminences, which in their turn ulcerate, and produce scars and furrows of the most unseemly kind. The cicatrices have often the appearance of an extensive burn. The progress of this eruption is generally slow and its duration protracted. It is always consecutive.

The fifth and last variety of the tubercular syphilides is not the least interesting of this species. It is often a primary symptom, and is distinguished from the preceding varieties by the flattened appearance of the tubercles, which are commonly, but erroneously, called "*pustules plates.*" This eruption is characterized by round, thick, and flattened tubercles, varying in size from that of a lentil to that of a shilling at their base, and of a vivid copper colour, and are perforated on the top by small linear ulcers. The smaller variety is found chiefly on the sides of the nose and lips, the larger on the scrotum, penis, pubis, thighs, and anus. The summit of the tubercle soon ulcerates, and presents the appearance of a narrow slit, from which a sanious fetid matter is discharged. The whole scrotum is sometimes covered by these tubercles; they are isolated, perfectly round, and very prominent. Round the margin of the anus they may coalesce and form large surfaces, but the ulceration is always superficial. The tubercular syphilitic eruptions are represented by several excellent and correctly coloured plates in M. Cazenave's atlas.

*Syphilitic papulæ.* These eruptions are characterized by small, solid, resistent elevations, of a well-marked copper colour, containing neither pus nor serum, and diffused over a considerable extent of surface. The papular syphilides manifest themselves in two distinct forms, one of which assumes an acute, and the other a chronic character. *Lichen syphilitica* is the acute variety, and is generally ushered in by slight febrile symptoms, as the exanthemata. It appears in the form of innumerable small papulæ, sometimes confluent, and possessing a shining appearance, which, joined to the copper tint, gives them a most remarkable aspect. This is the eruption which Carmichael says so frequently accompanies blennorrhagia. It occurs on most parts of the body, but especially on the neck and face, and, from the similarity of several of its symptoms with those of the febrile eruptions, it is not unfrequently mistaken for one of the latter.

The duration of this eruption is generally short, and it terminates by resolution in the course of a fortnight or so. The papulæ generally terminate in a kind of insensible exfoliation, leaving no other trace of their existence than slight stains, which soon disappear.

In the other variety the papulæ are much larger than those of the preceding, and commence in the form of small yellow blotches. They are isolated and scattered over a large extent of surface, and the eruption may be seen in different stages in the same individual at the same time. In one place we may find hard, prominent, copper-coloured papulæ; in another small cuticular elevations, more prominent, softer, and of a paler red. Here we observe yellow stains, approaching to a rosy tint, which are about to become papulæ; and elsewhere we see small gray spots, more depressed than the preceding, which are merely the traces of former papular elevations. All these are separated from each other by sound portions of skin, the colour of which, however, is of a peculiar *earthy* appearance, perfectly unique. This variety occurs most commonly on the extremities, shoulders, neck, forehead, and scalp. It is not accompanied by pruritus, is always consecutive, and frequently complicated with other venereal eruptions. It assumes almost invariably a chronic form, and disappears, sometimes after the lapse of several months, by resolution. The author relates several cases illustrative of this and the preceding variety.

*Syphilitic squamæ.* The scaly syphilitic eruptions are extremely interesting. The anatomical characters are essentially different from the preceding species; for, instead of the usual products of inflammation which characterize the diseases already described, we have here a double lesion of the chromatogenous and blennogenous apparatuses—those which secrete the colouring matter of the skin and the epidermic matter. These eruptions are characterized by dry, grayish, slightly adherent, epidermic lamellæ, covering irregularly-shaped patches of skin, slightly prominent and of a copper colour, and sometimes even of a blackish tint. The lamellæ are merely the results of a thickening of the epidermic matter, which dries, cracks, and is detached in scales. M. Cazenave describes three varieties of this species: syphilitic lepra, syphilitic psoriasis, and a *horny* variety which Biett mentions.

Syphilitic lepra must not be confounded with the *lepra venerea* of authors, which is a pustulo-crustaceous disease (*impetigo syphilitica confluens*), and another example of the mischief produced by vague terms. Syphilitic lepra appears in the form of circular discs, with depressed centre and raised edges. The patches, unlike those of lepra vulgaris, scarcely ever exceed the circumference of a shilling. They commence in the form of a small elevated patch of epidermis, not unlike a broad papula. They are first of a copper colour, and subsequently of a violet hue, and are covered with dry, hard, grayish scales, which fall, and are renewed incessantly until the disease is at length suppressed. It terminates by resolution, and leaves behind a dark circular stain, which corresponds exactly to the round disc which it has replaced. This eruption, which is one of the rarest of the syphilides, occurs generally on the limbs, and is preceded by some constitutional disturbance.

Syphilitic psoriasis is a much more common variety than the preceding. It appears in the form of P. diffusa, but more frequently in that of P. guttata. In the first the patches are broad, irregular in form, and of a deep coppery-red colour, and are covered by hard, brittle scales, of a dull white tint. This variety occurs frequently on the face. It sometimes coexists with the second form of the eruption, and then it is seen on the palmar aspect of the hands, and about the malleoli. Psoriasis guttata is much more commonly met with than the preceding form. It is characterized by prominent patches, without a central depression, of a round or oval shape, and rarely exceeding the size of a farthing in circumference. These patches are isolated, scattered in considerable numbers, of a vivid copper colour at first, which finally passes into a grayish or leaden hue, and are crowned with gray and very adherent squamæ. The scales, however, are very flimsy, are reproduced with difficulty, and the red elevation which they leave behind is quite pathognomonic of the disease. Its base is encircled at the point where it rises above the level of the skin by a small white rim, with a fringe or border (*lisière*), at first sight like that of a faded vesicle. After continuing for a certain time, the patches begin to disappear, and their colour at the same time becomes fainter, but the latter is of longer duration than the former. It changes successively from a copper tint to a brown, then to a gray, and finally vanishes altogether, leaving no trace of its existence behind. This eruption is generally diffused over the whole body.

The *horny* variety of the syphilitic squamæ is generally developed in the palms of the hands and soles of the feet. It appears in the form of slightly elevated spots of a copper colour, circular form, and covered by hard grayish scales. Sometimes these scales are multiplied in great numbers, and coalesce, so as to form one large thick patch, which increases by successive layers, cracks, and forms painful fissures. The squamous patch is invariably surrounded by a pretty broad copper-coloured areola. In another set of cases the patches do not exceed in circumference that of a farthing, are rounded, scarcely prominent, and have a hard, white, horny point in their centre, which penetrates like a corn into the substance of the skin. Around this horny matter we may constantly observe a circle about two lines in breadth, like a zone, and of a very peculiar colour. This variety may exist for months, and even for years, without occasioning much inconvenience.

The syphilitic squamæ are most commonly consecutive, and hence are generally met with in adults; but M. Cazenave has often seen these eruptions in young children, and even in new-born infants. In the latter, he has met with a case of this disease developed in the palms of the hands and soles of the feet.

M. Cazenave next proceeds to describe the *concomitant* symptoms of the syphilides, which may be developed in the skin itself, the cellular tissue, the appendages, the mucous and serous membranes, the osseous and fibrous tissues, and consist in blotches, tumours, ulcerations, caries, necrosis, &c., but want of space prevents our entering into the details of this interesting chapter. We must therefore be content with recommending it to our readers as well worthy perusal, and pass on at once to the causes and treatment of the syphilitic eruptions.

Causes. M. Cazenave denies that mercury is a common cause of syphilitic eruptions. He has administered that remedy in a host of cases at the Hospital of Saint Louis, where no symptoms of the kind were produced, and he has, on the other hand, repeatedly met with them in cases where mercury had never been administered. The result of his extensive experience in the treatment of these diseases goes to prove, that if the mercurial treatment will not invariably prevent the appearance of secondary symptoms, it certainly is not the cause of the syphilitic eruptions, whose real origin is a specific virus, involving the constitution generally. The syphilides may be primary, secondary, or hereditary. In the first instance, they are the immediate results of blennorrhagia or chancre, or they may appear consentaneously with either of these lesions. In the second, they are manifested long after the original disease has disappeared, in a system poisoned by the syphilitic virus, and only waiting for some accidental cause for their development. In the third, they are transmitted by the venereal act to the embryo, but the manner in which this is accomplished, and the laws which influence its transmission in one case more than another, are mysteries which baffle all inquiry.

Those of the syphilides which most frequently appear as primary, i. e. accompanying the earliest symptom of syphilis, are erythema and roseola syphilitica, syphilitic papulæ and vesiculæ, the lenticular variety of the syphilitic pustule, and superficial ecthyma.

The secondary, and most common form of the syphilides is always developed by some accidental cause, and frequently occurs after the lapse of an almost incredible period of time from the date of the primary and original disease. The author relates several cases, showing how the venereal eruptions were not developed for thirty and thirty-five years after the system had been primarily infected, and in a number of cases the primitive affection was blennorrhagia.

In 172 cases treated by the author, the average time that elapsed between the primary disease and the syphilides was ascertained to be five years and ten months, when blennorrhagia was the elementary form of the venereal complaint; four years and three months when they were the sequelæ of chancre; three years and one month when preceded by chancre and bubo; after repeated primary infections eight years and three months; after chancre, complicated with blennorrhagia or bubo, three years and eight months; and, as a general mean, five years and two months may be considered to be the period which elapses between the termination of syphilis and the invasion of the venereal eruptions. Chancre, complicated with bubo, seems to produce the syphilides more rapidly than any other of the primary symptoms, complicated or not, and the widest range is between chancre and blennorrhagia; five years is the shortest period for the development of the secondary eruptions by the former, and thirty-five years the longest by the latter. The tubercular syphilides are the longest of appearing after the primary disease, and the pustular syphilides the most rapid.

M. Cazenave does not agree with Mr. Carmichael that there is a certain degree of relationship between the nature of the primary symptom and the particular form of eruption which may succeed it; or, in other words, that certain primary symptoms will produce certain secondary lesions. The author maintains that any form of the syphilides may succeed indifferently blennorrhagia or chancre, in a severe or mild form. M. Cazenave also positively states, as the result of long and careful observation, that the venereal eruptions may and do appear as sequences of a simple blennorrhagic discharge, and in this opinion he is supported by MM. Martius, Legendre, and Baumes. Out of 157 cases which he selected and examined with the most rigid scrutiny, and in whom one or other of the syphilides existed, 42 had for their origin chancres, simple, or complicated with bubo; 60 had originated in blennorrhagia, with or without buboes; 48 had chancres, blennorrhagia, and buboes, together or at different periods; 5 had the bubo *d'emblée;* and 2 originated in the primary form of eruption; hence he concludes, that if blennorrhagia is not the most frequent cause of the syphilides, it certainly is as commonly so as chancre.

The eruptions which M. Cazenave has observed as the sequences of blennorrhagia were in the following proportions in 58 cases: Syphilitic tubercles in 23; S. pustules in 16; S. papulæ in 6; S. squamæ in 7; S. exanthemata in 2; S. vesiculæ in 4. The author's researches lead him to the conclusion that the primary disease occurs most generally between the ages of eighteen and thirty, and the syphilides between those of twenty and forty. He divides the syphilitic life (*la vie syphilitique*) into seven periods, and obtained the following results in 158 cases: Up to ten years only one case (hereditary); from 10 to 20, seven (2 hereditary); from 20 to 30, sixty-seven; from 30 to 40, forty-three; from 40 to 50, twenty-seven; from 50 to 60, eleven; above 60, two. With regard to the influence of the seasons on the development of the syphilides, the author considers that they occur more frequently in cold than warm weather. Out of 112 cases carefully examined by the author, 14 occurred in January, 12 in December, 11 in March, 9 in February, 8 in November, 7 in October, and the highest number in the warm months was 13, which occurred in June. These researches lead to a conclusion the reverse of that generally entertained upon the point, which the author himself admits. In alluding to the singular tendency which the syphilides have to appear upon the head, and especially the face, M. Cazenave states that in 172 cases, they occurred 66 times on the head exclusively; in 31 cases they appeared first on the head, and thence spread to other parts; and in 75 they attacked other parts of the body. Trade or profession has little or no influence upon the development of the syphilides. The preceding statements are supported, according to the author's general plan, by numerous cases, several of which are extremely interesting, and may be read with profit.

*Diagnosis.* The special character of the syphilitic eruptions, their contagious nature in some cases, and their destructive tendency, invest the diagnosis of these affections with a peculiar degree of importance. It would be equally unfortunate to mistake simple cutaneous diseases for these, as not to be able to recognize them when they really exist, and it is unnecessary to dwell on the frightful results sure to attend errors of this kind, examples of which we may see every day in the streets of this metropolis. The diagnosis of the syphilides is based upon a knowledge of their general characters, and on a certain *ensemble* which it is difficult to describe, depending on the peculiar colour and arrangement of the eruption and general state of the patient. M. Cazenave enters minutely into the diagnostic characters of each particular eruption; but as most of them may be found under the head of "Symptoms," which we have already pretty fully described, we must, for the present, be content with recommending this important chapter to the attention of our readers, and proceed to discuss the different therapeutic measures adopted by the author for the cure of the syphilitic eruptions.

### TREATMENT.

It were useless to enumerate the long list of remedies which have been employed in the treatment of constitutional syphilitic affections, we shall therefore confine ourselves to a consideration of those the utility of which has been demonstrated by experience. M. Cazenave gives a preference to mercury above all other remedies, in the treatment of the venereal eruptions.

He has found it, after long experience, to act with greater certainty and promptitude than any other medicinal agent in this class of disease, and although it may not be able to eradicate altogether the syphilitic constitutional taint, it can so modify it as to prevent a speedy relapse. Our own experience with regard to the treatment of the syphilides fully bears out M. Cazenave's views upon this point. We have witnessed in the practice of Biett, and more recently in that of M. Cazenave himself, the happiest results follow the judicious administration of mercury, and this after a variety of other remedies had failed either to remove or modify the eruption or the constitutional taint.

M. Cazenave strongly condemns, and justly so, the production of salivation, as recommended by some writers on this subject. He has found mercurial inunction most servicable in the secondary form of the syphilitic affections, in which the bones are involved, but still more so in the primary syphilides. A scruple at first, and subsequently two scruples of mercurial ointment may be rubbed in, and a warm bath every third day will cleanse the skin and facilitate the absorption of the mercury. It has been recommended to apply the ointment to the prepuce, but this practice is objectionable, in consequence of the irritation and tumefaction of the glans it produces. The author prefers applying the mercurial ointment internally in the form of pills, after the manner of Biett, and according to the following formula: Mercurial ointment and Sarsaparilla powder, of each three scruples; mix, and divide into forty pills. One to be taken morning and evening to begin with, and afterwards the dose may be increased to four pills during the day, but not to exceed this. This method, however, is slow, and unfortunately has a great tendency to produce ptyalism, which should always be prevented if possible.

*Protochloride of mercury.* Calomel is, perhaps, the least active of all the mercurial preparations in the treatment of the venereal affections of the skin; nevertheless, it has been recommended by Clare, Cullerier, and Brachet, of Lyons, and Biett has found it in some rare cases attended with good effect, when snuffed into the nostrils. It is now seldom employed.

*Bichloride of mercury.* This remedy ranks amongst the most useful preparations of mercury for the treatment of syphilitic skin complaints, but it is so difficult to be able to push it far enough to effect the object in view, and other preparations answering the purpose as well without this drawback, that practitioners are not fond of employing it frequently. Biett used to administer it in the form of pills, thus: Alcoholic extract of aconite, 6 grains; Bichloride of mercury, 2 grains; marshmallows powder, 18 grains; make eight pills. Begin with one pill *per diem*, and increase the dose gradually to four each day. For our own parts, we prefer using all remedies in skin complaints in a fluid form. They are more diffusible through the system in this than in the solid form, and we have invariably found their action more certain, and the curative effects more rapid when administered in the manner indicated. For this reason we prefer, with M. Cazenave, employing Van Swieten's liquor or Larrey's syrup to Biett's pills; besides, they are much easier borne by the patient. But M. Cazenave, like his preceptor, is generally fond of pills.

*Ammoniacal protonitrate of mercury.* This remedy, which is the soluble mercury of Hahnemann, is very useful. It is more easily managed and easier borne by the patient than the preceding. It operates with promptness, and may be used beneficially either in a mild or severe form of the eruption. The author prescribes it in the form of pills: Soluble mercury of Hahnemann, two scruples; Liquorice powder, two scruples; make forty pills. One pill night and morning, to be increased afterwards to four pills *per diem*. This remedy is borne well by feeble and delicate patients.

*Iodides of mercury*. Of all the mercurial preparations, and of all the remedies of whatsoever kind, which have been recommended in the treatment of syphilides, none can approach, in therapeutic value, the iodides of mercury. We are indebted to Biett for the introduction of these valuable remedies in the treatment of the venereal eruptions. This practitioner at first preferred the biniodide, and administered it in pills in the following form: Biniodide of mercury, ten grains; Liquorice powder, one drachm; make sixty pills. Dose, from two to three *per diem*. But he soon relinquished this preparation for the more manageable, and more efficient protoiodide of mercury. This is undoubtedly one of the most valuable remedies we possess, and it is certainly that under the influence of which we can almost invariably modify, if we cannot cure, the syphilitic eruptions. This agent seems to acquire a double value from the combination of iodine with mercury. In the great majority of cases it is borne easily by the patients, and may be continued for a considerable period without causing any inconvenience. It seldom occasions salivation. Like all the mercurial preparations it may derange the digestive organs, and occasion diarrhea; but these accidents occur but seldom, are slight in their nature, and speedily disappear on the temporary suspension of the medicine. The skin is specifically influenced by the protoiodide of mercury. The patches of disease assume a more lively and healthy aspect, and evince a tendency to resolution. But the beneficial influence of the remedy is not confined to the skin, for the general condition and aspect of the patient undergoes a remarkable alteration. The countenance becomes more animated, and the eruption advances towards resolution with a rapidity which, in some instances, is really surprising. It is worthy of note, that when the administration of the protoiodide is likely to be followed by beneficial results, these latter will begin to appear in the course of a very few days from the commencement of the treatment. M. Cazenave relates a number of cases in support of the remedial efficacy of the protoiodide of mercury in the syphilitic eruptions. He has not in the least exaggerated the merits of this excellent remedy, for we ourselves have seen all that he has said in favour of it fully borne out in practice, in his wards at the Hospital of Saint Louis, and in our own practice in this country. We cannot avoid giving in full the following remarkable case related by the author, in favour of this method of treatment:

"M——, aged 30, holding a situation in a public office, was admitted into the Hospital of Saint Louis on the 15th of June, 1834, to be treated for an aggravated form of syphilis, which had occasioned a general cachectic state of body. This patient enjoyed good health up to the age of 18, when he contracted a blennorrhagia, accompanied by chordee, which was cured by antiphlogistic measures. In the space of eighteen months after this he contracted chancres twice. In one case he was cured by the internal administration of calomel, and in the other by simple emollients. Since that period he has been attacked six or seven times by the same complaint, always at the base of the glans, and without concurrent ulceration of the mouth or throat. Six or seven years before his admission to the hospital he suffered from what he called an obstruction of the liver, accompanied by jaundice. Three years from his admission, and without any intermediate symptom, M—— was attacked by swelling and superficial caries of the frontal bone. These symptoms were treated by mercurial inunction, which occasioned violent gastric derangement. New tumours appeared upon different parts of the head, and disappeared without any treatment, and without occasioning injury of the bones. Soon after this the patient was obliged to perform a long and fatiguing journey from Strasbourg to Bayonne. Immediately after his arrival at the latter place he was attacked by severe pain in the head, congestion, and dyspnœa. The patient was bled freely, the original pains were removed, and a violent coryza ensued, unattended by inflammation of the nose. Emollient fumigations were applied to no purpose. The pain was renewed in an aggravated form, and thick scabs, accompanied by fragments of bone, were discharged through the nostrils, and at once declared the real nature of the disease. Three months after the appearance of the coryza a small pimple showed itself on the palate, broke, was converted into an ulcer, and spread rapidly. In two months the roof of the palate was perforated, and fragments of bone were discharged by the mouth and plates of the nasal bones through the nares. These symptoms disappeared under treatment by mercurial friction and Van Swieten's liquor, and the hole in the palate was partially filled up. A month afterwards the dyspnœa, congestion, and nasal hemorrhage were renewed, and small ulcerations appeared on the right ala of the nose and on other parts of that organ. The lesions of the palate and nasal fossæ reappeared and the right cheek was covered with tubercles, which were presently converted into irregularly-shaped ulcers, with sharp cut edges and grayish base. He now came to Paris, and was treated with Van Swieten's liquor, which only produced an exasperation of all the symptoms. Although his diet was confined to milk the constitutional disturbance continued, the disease proceeded with unabated violence, and the destruction of the palate was advancing rapidly. Iodine was administered for three months without the least benefit, but rather the reverse. The patient now entered the Hospital of Saint Louis in despair, without the slightest hope of cure, and only for the purpose of terminating, to use his own expression, a miserable existence, which he even repeatedly attempted to end by violent means.

"On his admission to the hospital he was emaciated, pale, his face hideous to look at, and where the skin remained intact it presented a yellowish colour. The whole of the nose, as far as the upper lip, was covered with thick yellowish-green scabs. These incrustations extended to the cheeks on either side, especially the right, and reposed on a well-marked copper-coloured base. A fetid sanious matter was discharged in abundance from fissures with which the scabs were intersected, and, on examination by the mouth, a horribly offensive smell issued from it, and the frightful ravages of the disease were brought into view. Almost the whole of the palate was gone, and the destruction appeared more extensive from the indented and furrowed edges it occasioned, and the gray malignant aspect of their borders. The left anterior portion of the superior alveolar case, deprived of teeth, was extensively destroyed, and presented the same kind of ulcerated surface as the exfoliated palate. The patient was moreover wasting from the effects of colliquative diarrhea, extremely irritable, prescribed his own treatment, and declared that he would not take mercury in any shape, to which he attributed his present unhappy condition. Nevertheless, Biett resolved to try the protoiodide of mercury, and, judging from former experience, with hopes of success.

"Accordingly, he deceived the patient by administering in the first instance small doses of opium, which were moreover of use in preparing the patient for the mercurial remedy. In a day or two the opium was omitted and the protoiodide administered for fifteen days. The patient himself was astonished at the marvellous effects of these pills, under the influence of which suppuration ceased, the scabs fell off, leaving a depressed surface of a coppery-red colour, slightly squamous, and possessing the characters of a solid firm cicatrix. The destructive process going on at the palate and maxilla was arrested, the aspect of these parts was entirely changed, and even presented some points of cicatrization. Such was the state of things when a modified form of cholera broke out in Paris, spread through the hospitals, and, amongst others, M—— was attacked, but not severely. The protoiodide, which he had taken for fifteen days only, was now suspended for eighteen days. Nevertheless, he continued to improve, although more slowly than when taking the pills. When he was scarcely convalescent from this complaint the patient was attacked by swelling of the face from cold, which terminated in erysipelas. As soon as this affection disappeared almost the whole of the scabs on the face fell off, the parts beneath cicatrized completely, as also the ulcerations in the mouth. During the month of August gastric symptoms supervened, and the medicine was discontinued until the end of that month. However, the patient took thirty of the pills without our knowledge, which he purchased from one of the patients in the ward.

"An attack of varicella, which lasted for six days, again interrupted the treatment, but when this eruption subsided the protoiodide was again administered and continued up till the middle of October, at which period M—— was completely cured. The nose was not destroyed, but it was covered with cicatrices of a round, depressed, whitish appearance, as were also the other parts of the face attacked. In the roof of the palate there was an irregularly-rounded cavity, about the size of a half-crown piece, with cicatrized edges, and forming a free communication between the mouth and nares. The left side of the upper maxillary bone was destroyed as far as the two last molars, which were the only teeth remaining on that side. The general health of the patient was good : he was getting fat, the colour returned to his cheeks, and the skin lost the sickly tint it presented before. A plate of silver and platina was fixed where the palate was perforated, and the patient was discharged perfectly cured."

The author relates a number of cases of a similar nature, treated with the same remedy, and with like success. He usually administers the protoiodide internally in doses of one, two, three, to four grains *per diem.* In the mild, simple forms, not of long standing, M. Cazenave uses the following formula :

Protoiodide of mercury, ten grains,
Liquorice powder, thirty grains.

Make twenty pills. Dose : to begin with one, to be increased to two, and afterwards to four pills in the twenty-four hours.

In the severe and inveterate forms of the disease, as, for example, the tubercular varieties, where a more active and energetic method of treatment is indispensable, the author prefers the following :

Protoiodide of mercury, two scruples,
Liquorice powder, four scruples.

Make forty pills. To be administered in the same manner as the preceding.

It is sometimes necessary to commence with two pills, and to increase the dose rapidly. The mercurial preparation should not be prescribed in too small doses. The author has repeatedly observed this remedy unattended by any beneficial results when so administered ; but, as soon as the dose was increased and given freely, it had the desired effect. Biett ascertained that when opium is given in combination with the protoiodide of mercury, the therapeutic qualities of the latter are completely neutralized: hence we should always prescribe it in an uncombined form.

Notwithstanding the great and important advantages to be derived from the mercurial preparations in the treatment of the syphilides, it is but fair to add that in some cases they do not succeed. Whether from idiosyncrasy, the patient will not bear the medicine long enough, or from a repugnance to it, he will not allow it to be prescribed, or from morbid irritability of the digestive organs, the remedy cannot be administered at all; from whichever of these causes it occurs, there is no doubt but we are sometimes baffled in our attempts to relieve the patient. Other remedies have been recommended when the foregoing fails, but our faith in their efficacy is not great.

*Acids.* In some of the milder syphilitic affections, as S. roseola, S. papula, the exhibition of the acids is not unfrequently followed with benefit. The author orders nitric acid (dilute) in doses of four minims, three times a day, in barley-water or orgeat. In the severer forms it may be tried when the preceding medicine fails.

*Gold.* The preparations of gold are not of much service in the secondary forms of syphilis, and the author has very little faith in their remedial powers in the treatment of the syphilides. The preparations of silver, so strongly recommended by M. Serres, of Montpellier, have been frequently tested by Biett and M. Cazenave in these complaints, but always without success. The author recommends sudorifics as useful auxiliaries to the mercurial preparations, and has even found them occasionally beneficial when unaided by other medicine. Iodine in its simple form, although extolled by many writers for its efficacy in syphilitic secondary diseases, has invariably failed in the hands of M. Cazenave. But there are combinations of iodine, other than those already described, which are extremely useful in many cases: these are the iodide of iron and iodide of potassium.

*Iodide of iron* has been recommended by M. Ricord in secondary syphilis, and the author's experience leads him to believe that it may be sometimes useful, but not so frequently as M. Ricord imagines. This preparation appears to us to be objectionable from the chemical changes which it is liable to, for it is almost impossible to keep it from undergoing a certain degree of decomposition in the solid form, and even the solution, with a coil of iron wire to preserve it, cannot be kept long undecomposed. In our own practice we use a *syrup* of the iodide of iron, which continues for several months without undergoing any change. This preparation is made by several London chemists.

*Iodide of potassium.* M. Cazenave has found the iodide of potassium to be only second to the iodide of mercury in its valuable therapeutic effects in the treatment of the syphilitic eruptions. Indeed, he seems to think that in some instances it is fully as efficacious as the mercurial preparation. Although he has occasionally observed it to cause considerable pain at the epigastrium and posterior fauces, it can generally be continued six or seven weeks, or longer, with impunity. The author uses two formulæ, a stronger and a weaker, which are prescribed according to the condition of the patient, the irritability of the constitution, and the duration and severity of the particular eruption present.

Iodide of potassium, ʒij;

Distilled water, ℥xvj;

Syrup ℥ij.

Mix. Dose: two or three spoonfuls per diem.

Or the following:

Iodide of potassium, ʒij;

Syrup ℥vj.

Dose: to begin with one spoonful, then two, and subsequently three in the twenty-four hours.

Such are the various modes of treatment which Biett and Cazenave have found to be attended with most benefit at the Hospital of Saint Louis, in the venereal diseases of the skin. As a general rule the author preferred the mercurial preparations to all other remedies, and only had recourse to the different remedial agents noted above, when he could no longer administer the protoiodide or in cases when it was inadmissible from the first. The powerful effects of this remedy are more strikingly displayed in the tubercular forms, and those complicated with tumefaction of the soft parts and periostosis, than in any of the syphilides. Its efficacy in removing those unsightly protuberances of both skin and bone which characterize the tubercular syphilides, is oftentimes truly wonderful. We have repeatedly seen them vanish, as if by magic, and were as much astonished at the rapidity as the completeness of the cure. While we have such an agent at our command, we do not despair of alleviating, if not curing, the most inveterate form of this hideous disease, provided the patient will bear the medicine; and our past experience justifies us in all that we have said in favour of this heroic remedy.

The practitioner must always be guided in the selection of the treatment by the nature and form of the eruption, its duration, the particular constitution of the patient, and the anterior treatment. The acids are indicated in the semiacute varieties, especially in S. exanthemata, S. vesiculæ, and one or two forms of syphilitic lichen. Sudorifics are mostly of use in the pustular, but particularly the squamous eruptions, and, as we have before stated, the mercurial preparations are specially applicable in the most severe forms of the syphilitic eruptions. Whatever may be the treatment adopted, it should always be preceded for a certain time by what we may call preparatory measures, both hygienic and medicinal. This observation applies in particular to those cases where the mercurial preparations are to be administered. Biett always gave opium in small doses for a week or two before he prescribed the protoiodide of mercury, and generally with good effect, and during the administration of the latter, he recommended the occasional use of the vapour-bath, and sudorifics.

M. Cazenave has not much faith in topical remedies in the treatment of the syphilides. He seldom uses ointments, unless to dress an ulcerated surface, and then employs this formula:

Protoiodide of mercury, ℈j;

Prepared lard, ℥j. Mix.

The author has sometimes employed with benefit the following ointment in cases of syphilitic lupus, with the view of modifying the parts of the skin:

Biniodide of mercury, gr. xij;
Prepared lard, ℥j. Mix.

The author has never found any benefit attend the use of escharotics, except in one form of syphilitic tubercle (*tubercule plat*) which he has occasionally modified by the application of nitrate of silver. In these cases aromatic vinegar has also been applied with advantage; but as a general rule escharotics are useless and frequently dangerous in the treatment of the venereal eruptions.

Baths are extremely useful auxiliaries in the treatment of the syphilides. The vapour-bath and douche, are particularly serviceable in the papular, tubercular, and squamous varieties. Tepid baths, rendered emollient by the addition of starch and gelatine, are beneficial in certain forms of the exanthemata, in lichen, and in impetigo syphilitica. Alkaline baths are also useful in these forms, and in certain stages of the pustular syphilides, when the dryness of the scabs seem to indicate that the ulcers are cicatrized. Cinnabar fumigations are often very serviceable in the tubercular eruptions, when administered directly by means of an apparatus to the diseased parts. It is peculiarly applicable in cases of tubercle on the scrotum and about the anus. Dr. Burgess recommends a preparation of iodine and sulphur in the form of vapour in these and similar cases:* ℞. Sulphuris ʒiij; Hyd. sulph. rubri ℈ij; Iodinii gr. x. M. ft. pulv. sex. Dr. Burgess states that he has found this remedy exceedingly beneficial in the squamous and tubercular eruptions. It should be applied in the form of vapour, by means of an apparatus, to the parts affected.

M. Cazenave considers it indispensably necessary that the treatment should be continued for some time after the eruption has disappeared. It is impossible to lay down any precise rules as to the limits of this period, which it is evident must be regulated by the character of the preceding disease and the tact of the physician. If the eruption was mild, of short duration, and yielded easily to the treatment, say in the course of a month or six weeks, it should be continued for a month longer, at the same time, gradually diminishing the dose. If the disease, on the contrary, was of a severe and obstinate character, of considerable duration, and the treatment had occupied a period of several months, the patient should be allowed to repose for about a fortnight after the disappearance of the disease, and then the treatment may be recommenced, and followed in the same manner as before for a given time, then discontinued and begun again as in the first instance. It is sometimes necessary to discontinue the medicine three different times before we have done. It is a singular coincidence, and one worthy of being remembered, that a patient who may have borne the mercurial remedies well for several months without intermission, will suddenly, and after a discontinuance of the treatment for a few days, evince an almost invincible intolerance of the remedy employed. This is a sure indication that the treatment is complete.

We have now concluded our analysis of the 'Traité des Syphilides.' The importance, as well as the novelty of the subject in its present form, has led us to give as copious a review of M. Cazenave's work as our limits would permit. Still we have not been able to do it the full amount of justice which it deserves. M. Cazenave is, beyond all dispute, the first dermatologist of the day. His works on cutaneous pathology are models of their kind, alike remarkable for their clearness of arrangement, lucid

* Translation of Cazenave's Manual of Diseases of the Skin; with Notes and Additions. By T. H. Burgess, M.D.

descriptions and practical character; and amonst these the monograph before us takes a prominent position. The 'Traité des Syphilides' is the first attempt that has been made to collect and publish in a separate and complete form the various syphilitic eruptions. It is, in fact, an elaborate extension of Biett's well-known article on the 'Syphilides,' published in the author's 'Manual of Diseases of the Skin,' enriched by the author's own experience, and copiously illustrated by cases and coloured plates. We could point to more than one English work on 'Diseases of the Skin,' which has received the approbation of a considerable portion of the medical press, for the excellence of the chapter on the Syphilitic Eruptions, which has been borrowed partly or wholly from the article on that subject in M. Cazenave's Manual. The dauntless hardihood and recklessness of consequences displayed by the author of one of these piracies forbids us to shut our eyes on one of the most heroic achievements in plagiarism that has, perhaps, ever occurred in the history of medical literature. The 'Compendium of Diseases of the Skin' by Dr. Jonathan Green, published as an original work, contains an article on the Syphilides which, with the exception of two or three lines at the beginning, is a *mere translation* of M. Cazenave's article. How any man, pretending to a literary reputation, or to a reputation of any kind, could have been guilty of such wholesale appropriation of another's property we cannot pretend to divine. But so it is; and there it will remain, a lasting stigma on the author, and on the press, which not only tolerated but lauded it.

We most cordially recommend the 'Traité des Syphilides' to those of our readers who are familiar with the language in which it is written. They can judge for themselves of the merits of the work from the analysis with which we have furnished them; and we have merely to say, in conclusion, that it has been our text-book in the treatment of the Syphilitic Eruptions for some time past, and we have always found it a safe and faithful guide in practice.

---

